# HOPE-AO overprescribing in older adults with chronic pain: a protocol for qualitative interviews with patients and healthcare professionals

**DOI:** 10.3310/nihropenres.14177.1

**Published:** 2026-07-29

**Authors:** Pat Schofield, Stuart G. Spicer, Richard Byng, Rupert A Payne, Ian Maidment, Phyo Kyaw Myint, Paul McNamee, Carrie Stewart, Mark Horowitz, Victoria Abbott-Flemming

**Affiliations:** 1 University of Plymouth School of Nursing and Midwifery, Plymouth, England, UK; 2 PENARC, University of Plymouth, Plymouth, Devon, PL4 8AA, France; 3 University of Exeter Primary Care Research Group, Exeter, England, UK; 4 Aston University, Birmingham, UK; 5 University of Aberdeen School of Medicine Medical Sciences and Nutrition, Aberdeen, Scotland, UK; 6 North East London NHS Foundation Trust, London, England, UK

**Keywords:** Pain, ageing, overprescribing

## Abstract

**Background:**

Pain in the older population is often poorly identified and subsequently poorly managed. Many myths and misconceptions exist amongst both older adults and healthcare professionals, including fear of using other methods of pain management and failure to monitor the impact of current approaches. This leads to underreporting of pain by the older adult population, overprescribing of pain medication, and failure to monitor the impact of prescribed drugs until major health crises such as falls or other serious complications occur, which can result in hospitalisation.

**Methods:**

The aim of this study was to explore chronic pain prescribing in older adults, both in terms of the population itself and in terms of individual perceptions, experiences and overall needs. As such, two work packages were employed, firstly to explore the wider prescribing patterns using the CPRD database (quantitative work package), and secondly to explore individual experiences using qualitative interviews (qualitative work package). The mixed methods methodology included an epidemiological approach along with qualitative methodology and a discrete choice experiment. Ultimately, the final outcomes of this investigation will lead to adopting a codesigned intervention which will be evaluated in a later research programme. This protocol focuses on both the qualitative work package and the wider project, including a brief outline of the quantitative work package. A more detailed technical protocol for the quantitative work package is available in a linked publication.

## Plain Language summary

People being prescribed too much pain medicine, for too long, and often when people don’t need it. Known as “overprescribing”, this is a particularly big problem in older adults. We need to solve overprescribing but are not yet sure about the best way of doing this.

Our project will investigate prescribing of pain medicine in older adults, to understand the issue and work towards a solution. There are two important things we need to look at; i) to understand more about the older adults who are taking pain medicines ii) to understand more about people’s experiences of taking pain medicines, along with their experiences of managing pain.

We will look at a big database of medical data, to understand more about who is taking these medicines, for how long, and what health conditions they have. We will interview 30 people who are taking these medicines, along with their family carers and 10 healthcare professionals. This will help us to understand people’s perception of how well these medicines work, how they feel about taking them, what else they do to manage their pain, and what they think about their wider healthcare.

We will develop a panel of ten people, overseen by a patient and public involvement lead. We have already worked with patients and members of the public in preparation for this project. The panel will meet monthly, and help with things like solving problems, sharing ideas and expertise, planning activities, and making sense of findings.

Towards the end of this project, we will bring together everything we have learned, and share what we have found out. We will prepare for a future project that follows on from this work, where we work with older adults with chronic pain, to design guidelines for better and safer pain management.

## Background

Epidemiological studies demonstrate that chronic pain is experienced by over 40% of UK community dwelling older adults,and is often poorly managed. ^1^ Ageing impacts upon the pain experience in ways that are not yet fully understood, with suggested mechanistic changes including, decrease in a-delta nerve activity, loss of proprioception, alteration in peripheral nerve activity, lower pain thresholds and hyperalgesia. ^2^ There is evidence of several painful conditions being more prevalent in older adults, including osteoarthritis, which results in joint pain. ^3^ Vertebral compression fractures are also very common. ^4^

About 95 million people across Europe are estimated to have chronic pain, ^5– 8^ compounded by inequalities in access to treatment. A large-scale survey across 15 European countries ^5^ showed high levels of pain medicine prescribing: NSAIDs (44%); weak opioids (23%); paracetamol (18%); and strong opioids (5%). Also, 40% of people experiencing pain have inadequate pain management. Whilst this survey focussed across all age groups, a recent overview by the International Association for the Study of Pain highlights disparities in effective pain management by age, along with sex/gender, ethnicity and socioeconomic status. ^9^ Our study aims to address these sociodemographic and cultural disparities, through a focus on older adults in two contrasting diverse areas (inland and coastal).

Overprescribing of pain medication is a substantial concern, and a key DHSC report ^10^ has highlighted the problem of overprescribing of dependence forming pain medications, including opioids and gabapentinoids. Overprescribing of opioids is particularly prevalent in older adults. ^11, 12^ Tertiary amine tricyclic anti-depressants are also often used for management of neuropathic pain, but are not advisable for older adults, because of side effects. ^13^ Management of pain in older adults presents many challenges, including a reduction in the effectiveness of medication in the long term and increased tolerance (which can make pain worse – e.g. opioid-induced hyperalgesia), along with multimorbidity and polypharmacy (prescribing of multiple drugs). ^14, 15^ The recent report by England’s Chief Medical Officer ^12^ suggests that overprescribing in older adults needs to be reduced, to enable improved quality of life, noting that this is the fastest growing population in society, with much to contribute within their families and communities. We therefore have a social responsibility to ensure older adults can contribute effectively as opposed to becoming increasingly frail, and losing dignity and independence. Myths and misconceptions surrounding pain and ageing are prevalent such as, “you get used to the pain”, “I expect pain at my age” and “older adults do not feel as much pain as their younger counterparts”. ^16^

In addition to overprescribing, analgesic medication it is often co-prescribed with other medications, such as psychotropic drugs used to treat psychological distress. Older adults are prescribed a median of seven different medications. ^17^ Polypharmacy (use of multiple medications) is particularly common amongst older adults and is strongly associated with increased mortality and morbidity. ^17^ Excessive polypharmacy (>10 medications) is associated with inappropriate medication use. ^17^ Notably, several medications are prescribed for both pain and distress (e.g. tricyclic antidepressants and gabapentinoids), and pain and distress syndromes (such as fibromyalgia and depression) together represent a range of related disturbances of brain-body functioning ^18^ that share underlying social causes including trauma and adversity. ^19^ Such medications share distinct but related mechanisms of tolerance and withdrawal. ^20^ A related issue is the link between chronic pain, poor sleep and low mood, with prescribing of multiple medications to manage this complex interaction of pain and distress. The European Pain Federation has issued a position paper on the use of these drugs in the general population, but no such paper has been produced for older adults. ^12^ There is therefore a strong rationale to consider wider co-prescribed psychotropic medication, when investigating issues around prescribing for chronic pain in older adults.

A review by Williams et al. ^21^ suggested that rational deprescribing of prescription drugs would reduce the risk of adverse reactions and hospitalisations. Other studies have reported positive benefits to older adults from deprescribing, including a 24% decline in mortality, a significant reduction in referrals to acute care, and reduced healthcare costs. ^22^ There have been some steps towards deprescribing and better prescribing of pain medication, and several tools have been developed, including FORTA, BEERS, and the STOPP/START criteria. ^23^ Another review ^24^ identified interventions including decision support tools, educational programmes, and medication reviews by pharmacists and/or healthcare providers. The authors concluded that deprescribing interventions using the STOPP criteria seem effective in reducing pain overprescribing and polypharmacy in older adults, by providing a physiological systems-based tool that defines clinically important issues relating inappropriate medications. However, further research is needed on the effectiveness of these approaches on clinical outcomes, and how to implement them most effectively. Furthermore, these frameworks fail to address patients’ preferences, which are key to cooperation and adherence, nor do they identify patients’ individual needs and plan care delivery accordingly. This is a major limitation that this study aims to address by using a person-centred approach, where patients are at the heart of planning and development.

Another factor that influences pain prescribing and reporting is the “7- minute consultation”. ^25^ Tai-Seale et al demonstrated that the time allowed for a GP consultation prohibits a thorough pain related discussion. Consequently, prescriptions are often quickly initiated and repeated. Developing a joint framework between healthcare professionals and patients would enable optimum use of the 7-minute consultation to ensure medication management meets the STOP/START criteria. ^26^ Additionally, the potential for simple changes to deliver impacts has been demonstrated on a small scale. For example, an approach in Somerset ^27^ has used a simple opening question (“show me your meds please”) during routine care visits to identify unnecessary and unneeded medicines, helping to reduce wastage and save resources – while also leading to more optimised prescribing for some patients. There has been very little research into older adults’ perceptions of taking medication, in addition to uncertainty about who to target – which this study will address. A survey ^17^ of 100 adults aged 65+ years regarding their medication intake revealed that 81% percent believe they take a large number of medications. Whilst 79% believed these are necessary, 85% would be willing to stop if recommended by their GP, 62% would be willing to take fewer medications, and 41% would want follow-up by a specialist polypharmacy clinic. This small study suggests that there is an appetite amongst older adults to reduce their medication intake, but more research is needed. Such decisions are complex and to some extent, both patients and professionals need to accept uncertainty in this process (e.g. SHERPA: a new model for clinical decision making in patients with multimorbidity. ^28^

A scoping review ^29^ highlighted how both patients and healthcare professionals were anxious about stopping medications, even though patients felt overwhelmed by the amount they were being prescribed. Using JBI methodology ^30^ the review identified 20 papers highlighting that deprescribing can be safe and acceptable to clinicians, but specific effects were very varied and patient views were often not reported (to be addressed in our study). The PPIE partners in this review highlighted that medicines represented more to the patient than just tablets, suggesting the need for person-centred prescribing. Such findings highlight the lack of theory underpinning deprescribing.

In summary, overprescribing of chronic pain medication is a pressing problem in older adults. There is limited research and limited understanding of person-centred approaches to pain management within this population, as well as a lack of understanding about the population itself. Such issues are compounded by poor medication monitoring and infrequent reviews. A combined caregiver–patient-centred approach should encourage collaboration between prescribers and pharmacists to reduce overprescribing in older adults. Reducing and stopping medicines encompasses many factors including: life expectancy; reduced survival prognosis; excluding medication with questionable evidence of effectiveness; and promoting medication prescriptions with favourable risk-benefit ratios. There is evidence that withdrawal of unnecessary medication has positive impact upon the quality of life in older adults, along with potential health economic benefits from reducing waste. However, the most appropriate framework for person-centred medication deprescribing, optimisation and pain management in older adults is yet to be established. To establish the focus of such a programme, we need to know more about the population and their varied concerns.

We therefore propose a study that explores the prescribing of drugs (including opioids, non-steroidal pain medication, low dose tricyclics, paracetamol and gabapentinoids) for chronic pain management in older adults (65+), along with experiences of pain symptoms, the wider healthcare system, and people’s priorities and preferences around pain management. We will adopt a person-centred approach for engaging with older adults, empowering them to be heard, whilst helping to develop potential solutions for better pain management. We will incorporate both quantitative and qualitative work packages (WPs), to better understand the older adult population who are taking medication for chronic pain, along with their experiences of prescribing and pain management – and the views of family carers and healthcare professionals. Interpretation of the results with our lived experience partners will allow us to determine the focus of future programme grant funding to work with older adults in co-designing a patient-centred prescribing and pain management framework, encompassing the attitudes and beliefs of patients and healthcare professionals.

## Aims and objectives

The aim of the study is to explore chronic pain prescribing in older adults, both in terms of the population itself, and in terms of their experiences and overall needs. This includes 1) developing a good understanding of analgesic prescribing patterns in the context of various clinical, therapeutic and sociodemographic factors, which will help identify which patients are most likely to benefit from targeted interventions as well as shaping potential intervention strategies, while 2) also learning about the perspectives of people prescribed these drugs, along with perspectives from carers and multidisciplinary team members, which will further help inform intervention design. This work will provide the foundation for co-designing a pilot intervention/framework within a future research Programme (see Future Work Plans) and identifying participants who will work with us as experts by experience. As outlined in the background and rationale, some of the key knowledge essential for this future programme is lacking. Our development project aims will provide this core knowledge. Several key objectives (Obs) will allow us to achieve these aims:

Ob1: Understand which sociodemographic (e.g. age [above 65+], gender, deprivation, rurality, ethnicity) and clinical (e.g. mental health, multimorbidity, co-prescribing) factors are associated with prescribing of key analgesic drug classes (i.e. weak opioids, strong opioids, NSAIDs, paracetamol, gabapentinoids, low-dose tricyclics), combinations of these drug classes, and changes over time. (WP1).

Ob2: Investigate the association between the aforementioned analgesic drug classes (including clusters of drug classes and temporal changes), and important clinical and health service outcomes (particularly adverse outcomes). (WP1).

Ob3: Identify alternative treatment solutions to reduce the use of unnecessary analgesic pain medicines through exploring older adults and their caregiver’s: acceptability of currently used analgesic pain medicines (and other pharmacological agents for mood or sleep aspects of chronic pain); their current use; their trust of different prescribing professionals; and their willingness to try and accept other pain management modalities (e.g, self-management, non-pharmacological, safer pharmacological options) (WP2).

Ob4: Identify the current barriers and facilitators for optimising, deprescribing, and preventing unnecessary prescribing of analgesic pain medicines for older adults through exploring the experiences of health professional prescribers managing medicines for older adults living with chronic pain in the primary care setting. (WP2).

Ob5: Identify potential engagement strategies, prior to support, which can aid the optimisation and deprescription of unnecessary prescribing of analgesic pain medicines through exploring older adults, their caregivers and health professional prescribers (primary care) understanding of analgesic pain medicine risks and their willingness to engage in the optimisation and deprescription of unnecessary analgesic pain medicines. (WP2).

Ob6: Develop a prioritised list of acceptable engagement strategies and alternative treatment solutions to reduce use of analgesics and increase use of self-management, non-pharmacological or safer pharmacological options to manage chronic pain which can be tested in a future intervention, using the accounts of key stakeholders (older people, their caregivers and health professional prescribers). (WP2).

## Methods

The **NIHR PPIE statement** emphasizes the importance of **Patient and Public Involvement and Engagement (PPIE)** in research. We have included PPIE throughout the development, design and delivery of the study as active advisors in their role as experts by experience.

This 18-month study will use separate, parallel quantitative and qualitative work packages (WPs), integrating the findings at the end to inform the design of a future programme grant. The first quantitative WP will use an analysis of the CPRD database, so that we can get a clearer understanding of patterns and relationships of chronic pain prescribing within older 65+ adults in England. The second qualitative WP will use interviews to explore the perceptions and experiences of older adults, along with informal carers, and healthcare professionals, including prescribers. The findings of these two work packages will stand alone as important contributions to the literature, but in the final months of the project, we will also integrate the findings of both into a plan for a future programme of research, to codevelop a framework for optimal prescribing and pain management.

Work package 1 (0–15 months).

The full technical protocol for this WP is being written up for separate publication. A summary is provided here. The aim of this WP will be to explore the patterns of prescribing of analgesics and related medicines amongst older adults (65+ years). This will produce invaluable insights into the extent of prescribing for pain, how this changes over time, factors associated with prescribing, and potential consequences of analgesic use. This will provide us with a better understanding of which patients are most likely to benefit from targeted interventions, as well as informing what potential future interventions might look like and which aspects of care they might address. Our PPIE panel will be consulted before, during, and after the WP1 analyses, to inform both our approach and our interpretation of findings.

**Dataset:** This aspect of the project will use data from the Clinical Practice Research Datalink (CPRD). The CPRD collects representative, real-world data on 18 million active patients from GP practices across the UK, including sociodemographic data and key coded information on diagnoses, administrative activity and prescribing. It is linked to a range of other healthcare data, providing a powerful longitudinal dataset, and is widely used for pharmacoepidemiology and health services research, evident in over 3000 peer reviewed publications. For this study we will access the data through Exeter University, overseen by Professor Rupert Payne who has extensive experience using the CPRD (including as a member of CPRD’s data quality advisory group). Access to the CPRD data has been made through the CPRD electronic Research Application portal (eRAP) and is covered by approved protocol #24 004610 which is summarised below.

**Study design:** An exploratory retrospective cohort study will be conducted, including the following analyses:

• Quantify rates of prescribing of current (1+ prescription in last 3 months) and chronic (6+ prescriptions in last 12 months, and also current) prescribing of key analgesic drug classes, including probabilistic clusters of key classes.
• Identify changing patterns over time (1-year previously and beyond), including clusters of temporal changes.
• Describe the association of long-term prescribing and the aforementioned prescribing clusters with sociodemographic, clinical and other prescribing factors.
• Describe the association of long-term prescribing and the aforementioned prescribing clusters with relevant clinical and service outcomes (including use of non-pharmacological therapies) over a 12-month follow-up period, allowing for effect modification by baseline sociodemographic and clinical factors.

Associations will be modelled using multivariable regression (logistic, Poisson, etc. as appropriate) with random effects to account for practice-level clustering.

**Study population**: Older adults aged 65+ on the study index date, registered with at least 12 months of prior data, and who have received at least one prescription for analgesia in the previous 12 months.

**Analgesia prescribing**: The following analgesic drug classes will be considered by the analysis: high strength opioids (eg Morphine); low strength opioids (eg Codeine); non-steroidal pain medication; paracetamol; low dose tricyclics; and gabapentinoids.

**Exposures and covariates:** Sociodemographic factors will include age category (5-year groups), sex/gender, Index of Multiple Deprivation, ethnicity, and rurality (including coastal status if possible). Clinical factors will include Cambridge Multimorbidity Score, relevant mental health (e.g. anxiety/depression, severe mental illness, substance misuse, LD), pain conditions (e.g. osteoarthritis, endometriosis), functional disorders (e.g. irritable bowel syndrome, fibromyalgia), and other risk factors (e.g. previous falls, respiratory disorders, CKD, exploration of past trauma (if adequately coded)). Other prescribing factors to be considered will include polypharmacy (drug count) and relevant specific co-prescribing (e.g. antidepressants, benzodiazepines, z-drugs). Established codesets from our existing resources, HDRUK or similar, will be used to minimise data preparation time.

**Clinical and service outcomes**: Service use will be assessed using GP consultation rates, hospital admission rates (HES) and referrals to key services (e.g. physiotherapy, pain clinics). Linked HES data for unplanned hospital admissions will be used to identify important, relevant adverse clinical outcomes (e.g. AKI, bleeding, delirium, falls). Adverse drug reactions will also be captured through relevant codes within both primary care and HES data.

### Ethical approval

Ethical approval for this phase will be obtained through eRAP: https://www.cprd.com/research-applications .

Work package 2 (0–15 months).

The aim of this WP is to gain a deeper insight into the experiences of patients and staff regarding prescribing of analgesic drugs in the older population and a deep understanding of the attitudes and beliefs of the older adults themselves in taking or stopping these drugs. Specifically we want to understand: how older people and their family carers would describe the effectiveness of their medicines; how they understand these medicines to work (what are the benefits for them e.g. does not help with pain but helps them sleep); how they interpret side effects/risks; and what they would like to happen in relation to their medicine management within primary care, particularly in relation to possibly deprescribing pain medication and trying alternative pain management options. Where applicable, we will also explore experiences around co-prescribed psychotropic medications, intended to manage chronic pain associated psychological distress. This will allow us to propose codeveloped solutions, that are acceptable and pragmatic to our stakeholders, to be used in a future intervention to optimise and/or deprescribe pain medicines.

## Methods

### Design

This WP will be carried out in two parts: 1) using qualitative methodology, semi-structured interviews will be conducted with older adults, family carers and healthcare professionals (HCP); 2) using quantitative methodology, a pilot Discrete Choice Experiment will be conducted with the same sample.

### Ethical approval

Ethical approval has been obtained from both HRA and University of Plymouth (ref: 344044). All participants included in the qualitative interviews will be provided with a PIS and required to sign a consent form before participation.

### Population and recruitment

Older adults (65+) living within the community and family carers (up to n = 30) and informed by data saturation) in two areas (Plymouth and Birmingham), ensuring participants represent those living in coastal and inland locations along with representing minority ethnic communities. Healthcare professionals involved in prescribing to older adults in primary care settings, including GPs, Nurses, specialists and Pharmacists working within the same geographical regions, Plymouth and Birmingham (up to n = 10 and informed by data saturation) recruited through our professional networks.

Approximately half of our older adult/caregiver participants (n = 15) will be recruited through primary care settings in the two study areas. The Agile team will approach the GP practices within the two areas and identify and consent our participants. The other half (n = 15) will be recruited through existing contact networks and adverts along with the NIHR “Be part of Research database”. We will also make use of contacts within regional equality groups and PPIE and patient groups to make a strong effort to recruit older adults and caregivers from seldom heard groups. These participants will volunteer through social media and will be consented by the research team. We will recruit approximately half (n = 5) of our practitioners from each geographic area, again this has been agreed with the Agile team.

Analgesics, a group of medicines, are used for common pain conditions including Musculo-skeletal pain, arthritis and joint pain. We have defined analgesics as:

1. Opiates e.g., codeine, dihydrocodeine including in combination products e.g., co-codamol, co-dydramal.
2. Paracetamol
3. NSAIDs e.g., ibuprofen, but excluding low-dose aspirin for stroke prevention.
4. Low dose tricyclic anti-depressants (e.g., amitriptyline).
5. Gabapentinoids (e.g., pregabalin and gabapentin)

### Part 1 – Semi-structured interviews

Older adults and caregivers: An in-depth semi-structured interview guide will be developed using the literature and excerpts from the PATD questionnaire which was developed and validated to explore attitudes and beliefs of older adults. This 15-item questionnaire was developed and tested in the US with older adults and includes questions such as ““If my doctor said it was possible, I would be willing to stop one or more of my regular medicines” and “I would like to reduce the number of medicines I am taking”. However, some of the questions regarding costs of medication would not be included as they are not relevant to the UK setting.

Inclusion (Older Adults)

• Adults over the age of 65 years taking any of the prescribed medication groups for pain.
• Older Adults able to consent under the Mental Capacity Act 2005
• Adults over the age of 65 years who are able to understand and speak English.
• Adults over the age of 65 years who are not experiencing moderate to severe dementia
• Adults over the age of 65 years who are not terminally ill or receiving palliative or end of life care.
• Adults over the age of 65 years who are willing/able to sign the consent form to take part and do not meet the criteria of the Mental Capacity Act (2005).

### Inclusion criteria (Informal carers)

• Any adult living with and or supporting an older adult experiencing chronic pain.
• Any adult able to speak and understand English language
• Any adult willing to sign a consent form to take part in the study.
• Adults able to consent under the Mental Capacity Act (2005)

### Exclusion criteria (Older adults)

• Adults over the age of 65 years who are unable to understand and speak English.
• Adults over the age of 65 years who are currently experiencing moderate to severe dementia.
• Older Adults unable to consent under the Mental Capacity Act 2005
• Adults over the age of 65 years who considered to be terminally ill.
• Adults over the age of 65 years who unable sign the consent form according to the criteria of the Mental Capacity act (2005) to take part or unable to take part in an online or face to face interview.
• Adults over the age of 65 years who are not currently taking any of the listed medications for pain.

### Exclusion criteria (Informal carers)

• Adults unable to consent under the Mental Capacity Act 2005
• Adults unable to speak and understand English language
• Adults unwilling to take part in the current study

Healthcare professionals: The second set of interviews will be conducted with healthcare professionals working in primary care in the same two geographical areas, using the Theoretical Framework of Acceptability (TFA). ^30^ The 7-dimension TFA model explores: Affective attitude (how an individual feels about the intervention); Ethicality (how the intervention fits with the individuals values); Intervention coherence (the extent to which individual understands the intervention and how it works); Burden (the perceived amount of extra effort to participate in intervention); Perceived effectiveness (the extent to which intervention is expected to achieve its intended purpose); Self-efficacy (the participants confidence that they can perform behaviours necessary to participate); and Anticipated opportunity costs (the extent to which benefits, profits or values must be given up to engage with intervention).

### Inclusion criteria (Health care professionals)

• Health care professional responsible for prescribing, administering or issuing (including dispensing) any of the medications on the list.

Health care professionals willing to take part in the study.

### Exclusion criteria (Health care professionals)

• Healthcare professionals not currently prescribing administering or issuing (including dispensing) any of the medications on the list.
• Healthcare professionals not currently working with older adults.

### Analysis

All interviews will be conducted either face to face or online according to participants preferences and recorded and transcribed verbatim ensuring full anonymity for the participants. We will analyse the interview data using framework analysis. (All consent forms, information sheets and interview guide are stored on UK data service: REF: 15555).

### Right to withdraw

All participants have the right to refuse to participate or withdraw from the study without giving a reason before, during, and after the study. Participants who refuse or withdraw from the study will not be affected by the treatment or care received. Participants who withdraw from the study have the right to ask for their information to be removed. However, if their information was already involved in the analysis, the data would not be excluded.

### Debriefing

The researcher (CI) will inform the participants regarding the interview process and remind them that they have the right to refuse to answer any questions or withdraw from the interview or study at any time. Participants will also be informed about their right to ask for pauses and breaks or to terminate the interview at any time. If a participant indicates or expresses signs of distress during the interview, the CI will take a break immediately, ask the participants to terminate the interview and remind them of the right to withdraw from the study. In that case, the CI will offer participants professional health support by signposting to their GP and will offer additional sources.

### Interviewer bias

Interviewer bias can arise during and after the interview in the data analysis process. This personal bias will be minimised with reflexivity during the study’s planning, conducting and analysis. This will be accepted with personal and professional bias impacting the study. An interview guide with open-ended questions and prompts will be applied to focus on the participant’s experience rather than the interview’s interest. The interview guide has been developed based on our literature review on the topic and will be shared with our Advisory Panel for their comments.

### Recall bias

Participants will be asked to recall their experiences when they or their relatives were unwell. The time between the event and the interview can cause inaccurate recall or partial missing information. Also, participants’ emotions during that time may skew their responses.

### Emotional risk

Involvement in the study through interviews might distress the participants while sharing their experiences. There is a slight possibility of being upset because of remembering their experiences. Emotional upset is an understandable response, and their wellbeing is the priority. If participants do not feel well, they can interrupt the interview at any time or withdraw from the study without providing any reasons. These rights will be reminded to each participant before the interview. The participants who express or show distress will be supported after the interview. The CI will signpost to professional health support GP and ensure that these concerns are formally reported.

### Breaching of privacy

Participants may share personal or family information while expressing their experiences during the interview, which can compromise privacy. To maintain confidentiality, the CI will remind all participants about confidentiality and pseudonymised transcription in case of a breach of privacy.

### Risk to researcher

The researcher may be at risk of emotional stress due to being immersed in a lived experience during the interview. To address this potential issues, the researcher will arrange a debrief with an experienced supervisor. Additionally, the researcher will follow the University of Plymouth Lone Working Policy if interviews are scheduled to be conducted at participants’ homes or at a mutually agreed location.

### Part 2 - Discrete choice experiment (Health economics)

A Discrete Choice Experiment (DCE) will be piloted with both adult/carer participants and health care professionals. This will test the extent to which participants are able to complete a choice-task involving the attributes within the TFA model. ^30^ This will inform the design of a future larger preference elicitation study using DCE methods, to be implemented in a programme grant where a larger sample size would be recruited (n = 200+). Information from the DCE will give us an indication of the extent to which patients and professionals are willing to change medication-taking and prescribing behaviour, which are the most important barriers, and whether some groups are more willing to change than others. To inform the design of the pilot DCE, we will draw on prior information provided by the literature review being conducted to inform the interview topic guides that is considering existing evidence gathered around patient and care professional preferences for pain management.

### Synthesising both work packages (12–18 months)

In the final stages of the project, we will finalise the analyses and write up both work packages and bring our findings together in a synthesis that includes PPIE consultation. As part of this work, we will develop a list of PDG Competition 39 Reference: NIHR208337 Date submitted: 21 May 2024 Page 15 of 66 acceptable engagement strategies and alternative treatment solutions to reduce the use of analgesics and increase use of self-management, non-pharmacological or safer pharmacological options to manage chronic pain. We will also provide clear guidelines on who this can be targeted towards, i.e. who is most likely to benefit within the older population. In a future programme, these treatment solutions will be co-developed into a formal framework, and pilot tested in an appropriate cohort, incorporating the input of key stakeholders (older people, their caregivers and health professional including prescribers). Whilst it is too early to specify aspects of the future framework/intervention, we have identified key logical steps to address during the synthesis:

• Who to identify proactively (WP1 will help target) and gauge readiness to change.
• How to engage.
• Range of beliefs about pain/meds/solutions.
• Range of options that might make up a future intervention.

During this final stage, we will also build a network of engaged older people, caregivers and primary care healthcare professionals who are willing to assist with the co-development of this future framework of optimal prescribing and pain management.

Ethical approval has been given by Solihul HRA/REC IRAS ID: 344044.

### Patient & public involvement

We have an active PPIE member who has been involved in the development of the application to the funder and is employed by the university for the duration of the study. She will lead on the advisory panel, supported by members of the research team and she will be invited to attend the monthly team meetings. Our advisory panel will consist of 10 members of the public, some of whom were involved in previous applications. We are planning to provide a training workshop for this membership prior to commencement of the study to ensure they are aware of some of the “research terminology” that will be discussed during the project.

### Implications

We anticipate that this study will provide a baseline on which further work will be developed. From our collaboration with PPIE and Health care professionals we will initiate a codesign group who will use the data collected to develop the intervention for future evaluation.

## Data Availability

Consent, PIS and Interview guides along with Gantt chart submitted to UK Data Service ReShare Collection http://doi.org/10.5255/UKDA-SN-858429 Licence to CC-BY. No data are associated with this article.
