# Supplementary material for: HOPE-AO overprescribing in older adults with chronic pain: a protocol for qualitative interviews with patients and healthcare professionals
Source: NIHR Open Res. 2026 Jul 29;6:110. [Version 1] doi: 10.3310/nihropenres.14177.1 (PMC13560863; doi:10.3310/nihropenres.14177.1)
Supplement: Peer review history

# Reviewer report: Reviewer response for version 1

**DOI:** 10.3310/nihropenres.15431.r41383

**Published:** 2026-08-18

**Authors:** Margaret Dunham

**Affiliations:** 1 Edinburgh Napier University, Edinburgh, UK

Thank you for the invitation to review this publication of the HOPE-AO Protocol. This is a very good paper, it provides a valuable and informative detailed methodology on an important topic, and I look forward to seeing future publications detailing the PPIE involvement in WP1.

 I have only minor observations for the authors to note.

 The **Abstract **is clear, one minor comment is the tenses used, here and later in the document, these should be consistent with whether this part has been completed or is yet to commence.

 The **Plain Language Summary **is well presented. Another minor point is are the numbers  here the same as later in the protocol as 30 and 10 are noted here and later the numbers vary.

 The **Background **is clear and supports the proposal. Could all acronyms/abbreviated forms be introduced or explained with a footnote or similar?

 The **Aims & Objectives** section, there is possibly a little repetition here and this could  be more succinct as much is detailed in the later **Work Package** description.

 In the **Methods** section (page 8) should the term opioids be used in preference to opiates?

 The approach to recruitment and consideraition of potential bias is detailed and appropriate.

Is the study design appropriate for the research question?

Yes

Is the rationale for, and objectives of, the study clearly described?

Yes

Are sufficient details of the methods provided to allow replication by others?

Yes

Are the datasets clearly presented in a useable and accessible format?

Yes

Reviewer Expertise:

Pain in Older People, Qualitative Research, Ageing

I confirm that I have read this submission and believe that I have an appropriate level of expertise to confirm that it is of an acceptable scientific standard.

# Reviewer report: Reviewer response for version 1

**DOI:** 10.3310/nihropenres.15431.r41387

**Published:** 2026-08-03

**Authors:** christopher eccleston

**Affiliations:** 1 The University of Bath, Bath, UK

Abstract. The argument of under-reporting of pain and over-prescribing of analgesics is odd, as you might expect under-reporting to lead to under-prescribing. Perhaps it is drawn from two different literatures, but put together it looks odd.

 Background: The field is moving more towards a consideration of the impact of pain as well as its chronicity. The use of large numbers such as 40% runs the risk of trivializing pain as being part of life. It is really the 20% of the 40% who have high impact pain and are complexly disabled who we see presenting to health care.

 The polypharmacy issue is very well made, and a significant problem to be addressed.

 Methods: Missing for me are questions about performance. I understand that there is an interest in attitudes and beliefs, which is important, but I would start with:  1. Who prescribed these, 2. What do you think these are for? 3. Do you take them 4. Do they work, 5 How do you know if they work/don't work. 

 There is an increasing interest in the field in defining 'treatment-resistant' chronic pain, and to do that we need data on use and effect (Which has now been added, for example, to the UK Biobank).

 A small point, but I would make it 65 years and older, rather than 66 onwards (over 65) 

 The idea of Interviewer bias does not sit comfortably as it is probably not possible to be unbiased, so perhaps it is about Positionality.

 I have difficulty with the term 'upset' as it is not a psychological term. Perhaps one could be more exact.

Is the study design appropriate for the research question?

Yes

Is the rationale for, and objectives of, the study clearly described?

Yes

Are sufficient details of the methods provided to allow replication by others?

Yes

Are the datasets clearly presented in a useable and accessible format?

Yes

Reviewer Expertise:

Pain, Psychology, ageing

I confirm that I have read this submission and believe that I have an appropriate level of expertise to confirm that it is of an acceptable scientific standard.
